# Amelioration of premature aging in Werner syndrome stem cells by targeting SHIP/AKT pathway

**DOI:** 10.1186/s13578-025-01355-4

**Published:** 2025-01-25

**Authors:** Hei-Yin Tam, Jiaxing Liu, Tsz-Ching Yiu, Adrian On-Wah Leung, Chang Li, Shen Gu, Owen Rennert, Boxian Huang, Hoi-Hung Cheung

**Affiliations:** 1 School of Biomedical Sciences, Faculty of Medicine, The Chinese University of Hong Kong, Hong Kong S.A.R., China; 2 Centre for Regenerative Medicine and Health, Hong Kong Institute of Science and Innovation, Chinese Academy of Sciences, Hong Kong, China; 3 Eunice Kennedy Shriver National Institute of Child Health and Human Development, National Institutes of Health, Bethesda, USA; 4 State Key Laboratory of Reproductive Medicine, Nanjing Medical University, Nanjing, China; 5 Key Laboratory for Regenerative Medicine, Ministry of Education, School of Biomedical Sciences, Faculty of Medicine, The Chinese University of Hong Kong, Hong Kong S.A.R., China

**Keywords:** Werner syndrome, AKT signaling, SHIP, Senescence, WRN, Mesenchymal stem cell, Stem cell aging

## Abstract

**Background:**

Pathogenic or null mutations in *WRN* helicase is a cause of premature aging disease Werner syndrome (WS). WRN is known to protect somatic cells including adult stem cells from premature senescence. Loss of WRN in mesenchymal stem cells (MSCs) not only drives the cells to premature senescence but also significantly impairs the function of the stem cells in tissue repair or regeneration.

**Results:**

In this study, we profiled the signaling pathways altered in WRN-deficient MSC and applied pharmacological method to activate the AKT signaling in these cells and examined their cellular phenotype related to aging. We found that the AKT signaling in WRN-deficient MSCs was significantly suppressed while the AKT upstream phosphatases (SHIP1/2) were upregulated. Knockdown or inhibition of SHIP1/2 could ameliorate premature senescence in WRN-deficient MSCs. Moreover, SHIP inhibition stimulated MSC proliferation and suppressed expression of pro-inflammatory cytokines IL-6 and IL-8. The stemness of WRN-deficient MSC was also improved upon pharmacological treatments with the inhibitors.

**Conclusions:**

These results suggested that targeting the SHIP/AKT signaling pathway is beneficial to WRN-deficient stem cells and fibroblasts, which might be applied for improving the trophic function of MSC in, for instance, promoting angiogenesis.

**Supplementary Information:**

The online version contains supplementary material available at 10.1186/s13578-025-01355-4.

## Background

Werner syndrome (WS) is a classic progeria-like genetic disease that displays premature aging and a higher risk of cancer [1, 2]. Pathogenic mutation in *WRN*, a member of the RecQ subfamily of DNA helicases, is a cause of the disease [3, 4]. WS is classified as an autosomal recessive disorder in which pathogenic variants of *WRN* usually lead to complete loss (null mutation) or mislocalization of the functional helicase [5, 6].

WRN has been revealed to play an important role in the nucleus related to different processes of DNA metabolism, such as DNA repair, replication, transcription, and telomere maintenance [7]. WRN-deficient cells, including dermal fibroblasts, embryonic or adult stem cells, and even cancers, show lower survival with increased apoptosis and/or premature senescence [5, 8]. The role of WRN in stem cell survival and function is mainly observed in adult stem cells derived from mesenchymal lineages such as mesenchymal stem cells (MSCs) [9, 10]. Both embryonic stem cells (ESCs) lacking WRN and WS fibroblast-derived induced pluripotent stem cells (iPSCs) are reported to maintain pluripotency with high telomerase activity. However, MSCs differentiated from *WRN*^−/−^ ESC or WS iPSC and primary MSCs with WRN knockout (KO) or knockdown (KD) all exhibit accelerated senescence, decreased replicative potential, and loss of heterochromatin, suggesting that the loss of WRN leads to alteration in intrinsic pathways [9–11].

As stem cells rely on signaling pathways to maintain their self-renewal and stemness, we ask whether the premature aging phenotypes in WRN-deficient MSCs are linked to the alteration in signaling pathways that control cell senescence, proliferation, and survival. WRN has been known to participate in multiple pathways. Perhaps the most understood pathways are the DNA repair pathways such as non-homologous end joining, homology-dependent repair, and base excision repair [12]. Moreover, WRN interacts with p53, and p53-mediated apoptosis was attenuated in WS cells [13]. In *Wrn*-knockout mice, increased mortality was observed under the p53-null condition [14]. Given that p53 is a tumor suppressor, the p53-mediated pathway may explain the high incidence of neoplasms in WS patients. WRN participates in both the DNA repair and p53-mediated pathways in response to DNA damage and is thus vital for protecting genome stability. DNA damage response pathways include that of p38MAPK, which is hyperactive in WS fibroblasts. p38MAPK inhibition can suppress senescence and the senescence-associated secretory phenotype (SASP) [15, 16]. In addition to p38MAPK inhibitors, compounds that target the mTOR pathway can prevent premature aging in progeroid cells [17, 18]. Moreover, impaired mitophagy and NAD^+^ depletion were demonstrated in WS cells, indicating that restoring healthy mitochondria through NAD^+^ replenishment may be another approach to aging prevention [19].

The aforementioned pathways do not function independently. They may be related and cooperate to prevent premature aging in normal cells. For instance, the DNA repair pathways are related to the p53 and p38MAPK pathways, whereas mitophagy is related to the activations of mTOR and autophagy pathways in response to cellular stress. In this study, we focused on the AKT pathway, which is pivotal in promoting stem cell survival, proliferation, migration, cytokine production, angiogenesis, and differentiation [20]. We found that AKT activation was dampened in WRN-deficient MSC. Knockdown or inhibition of the upstream regulator (SHIP) could reactivate AKT signaling and stimulate the cells to proliferate faster and suppress senescence. Inhibition of SHIP could also stimulate MSC to produce more HGF and express less pro-inflammatory cytokines. Moreover, SHIP-inhibited MSCs could be better differentiated into chondrocytes, osteocytes, and adipocytes. These findings collectively illustrated the importance of suppressing the SHIP pathway to reactivate the AKT signaling in WS stem cells, which may provide insights into the potential treatment for WS.

## Results

### Downregulation of AKT pathway in WRN-deficient MSC

**Fig. 1:**
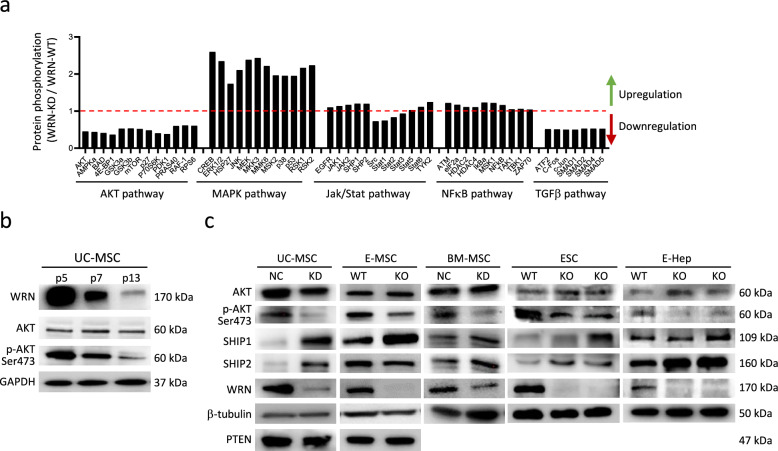
Downregulation of AKT pathway in WRN-deficient cells. **a** Comparison of protein phosphorylation in AKT, MAPK, Jak/Stat, NFκB and TGFβ signaling pathways by ELISA-based Protein Phosphorylation Array. **b** Decreased p-AKT and WRN during replication-induced aging in UC-MSC. **c** Western blot analysis of AKT phosphorylation (p-AKT, Ser473) and the upstream phosphatases SHIP1 and SHIP2 levels in WRN knockdown (KD) and knockout (KO) cells. p: passage number; E-MSC: ESC-induced MSC; E-Hep: ESC-induced hepatocyte; UC-MSC: umbilical cord-derived MSC; BM-MSC: bone marrow-derived MSC

To identify signaling pathways dysregulated in WRN-deficient MSC, we generated WRN knockdown (WRN-KD) MSC in human umbilical cord-derived MSC (UC-MSC). We screened the five major protein phosphorylation pathways which include MAPK, AKT, Jak/Stat, NFκB and TGFβ pathways using ELISA-based protein array. We found that AKT and TGFβ pathways were generally downregulated in WRN-KD MSC, whereas MAPK pathway was upregulated (Fig. 1a). Downregulation of AKT phosphorylation (p-AKT^S473^) was not only observed in WRN-KD MSC, but also in serially passaged normal MSC, in which WRN protein showed a decreasing trend when the cells had accumulated replication (Fig. 1b).

Upstream to the AKT signaling, PTEN (phosphatase and tensin homolog) and SHIP (SH2-containing inositol 5'-phosphatase) dephosphorylate PIP3 and thus negatively regulate the PI3K/AKT axis [21]. We found that both SHIP1 and SHIP2, but not PTEN, were upregulated in WRN-KD MSC (Fig. 1c). We also analyzed these proteins in ESC-derived MSC (E-MSC). WRN-KO ESCs (*WRN*^–/–^) was differentiated to E-MSCs, and these cells also displayed reduced p-AKT and increased SHIP1 (SHIP2 was not increased in E-MSC). SHIP1 (*INPP5D*) and SHIP2 (*INPPL1*) mRNAs were consistently augmented during cell culture for different types of MSC analyzed (Supplementary Fig. 1). Other cell types, such as ESC and ESC-derived hepatocytes (E-Hep) also showed reduced AKT phosphorylation and SHIP1 upregulation (Fig. 1c). These results suggested that AKT signaling pathway was commonly downregulated in WRN-deficient cells.

### Knockdown of SHIP in WRN-deficient MSC reactivated AKT phosphorylation and suppressed senescence

**Fig. 2:**
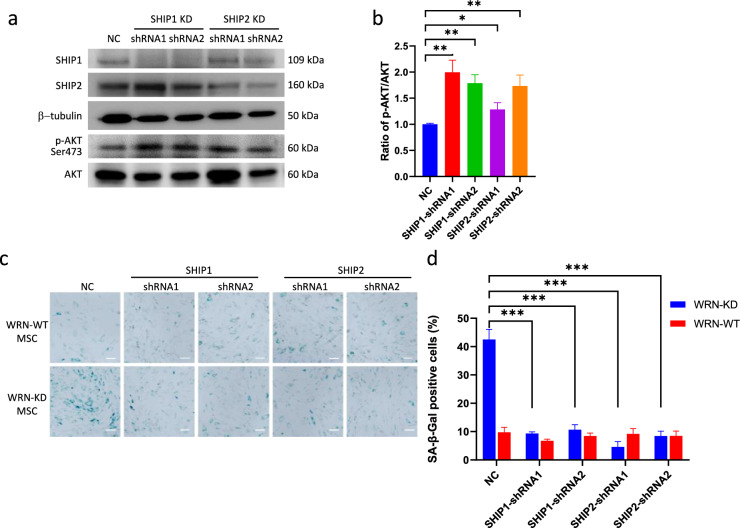
Knockdown of SHIP1 and SHIP2 in WRN-deficient MSC reactivated AKT phosphorylation and suppressed senescence. **a** Western blot analysis of SHIP1, SHIP2 and p-AKT and AKT (total) protein levels in SHIP1 or SHIP2 knockdown MSC. Two independent shRNA sequences were tested. **b** Quantification of p-AKT/AKT level in SHIP1/2 knockdown cells. **c** SA-β-gal staining for senescence in SHIP1/2 knockdown MSC (at passage number 6). **d** Quantification of SA-β-gal staining. *: *p* < 0.05; **: *p* < 0.01, ***: *p* < 0.001 (by two-sided unpaired Student’s *t*-test). Scale bar: 200 μm

To determine whether SHIP upregulation is related to AKT signaling downregulation, we knocked down SHIP1 and SHIP2 in WRN-deficient MSC respectively. As a result of SHIP protein depletion, p-AKT protein was increased accordingly, whereas total AKT (AKT_total_) remained unchanged (Fig. 2a). The ratio of p-AKT/AKT_total_ was increased following knockdown of each SHIP target, indicating an activated AKT signaling in the SHIP depleted cells (Fig. 2b). Next, we asked whether knockdown of SHIP could rescue senescence, a premature aging phenotype due to WRN deficiency in WS cells. As expected, WRN knockdown resulted in higher senescence-associated β-galactosidase (SA-β-gal) activity. Knockdown of SHIP1 or SHIP2, despite the deficiency of WRN protein, suppressed SA-β-gal activity in WRN-KD MSC (Fig. 2c, d). These data suggested that the upregulation of SHIP is linked to the premature aging phenotype in WRN-deficient MSC.

### Inhibition of SHIP activated AKT signaling and stimulated cell proliferation and clonogenicity in MSC

**Fig. 3:**
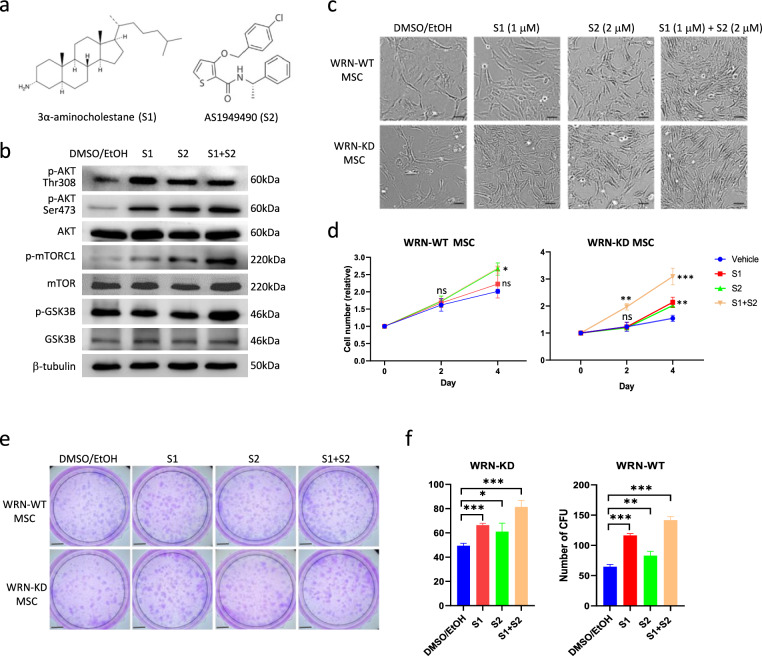
Inhibition of SHIP1 and SHIP2 activated AKT signaling and stimulated cell proliferation in WRN-deficient MSC. **a** Chemical structures of SHIP1 inhibitor 3α-aminocholestane (S1) and SHIP2 inhibitor AS1949490 (S2). **b** Treatments with either 1 μM of S1 or 2 μM of S2 or both activated phosphorylations of AKT (Ser473 and Thr308) and the downstream target mTORC1. **c** Bright-field images of MSC treated with S1 and S2. **d** Quantification of cell number during 4 days of inhibitor treatments. **e** CFU-F assay for the clonogenicity of MSC. **f** Quantification of colony numbers. *: *p* < 0.05; **: *p* < 0.01, ***: *p* < 0.001, ns: not significant (by two-sided unpaired Student’s *t*-test). Scale bar: 200 μm for (**c**) and 5.8 mm for (**e**)

Our previous study indicated that PI3K inhibitor (LY294002) significantly suppressed proliferation of normal (*WRN*^+/+^) MSC and compromised the pro-wound healing function [22]. However, LY294002 had little inhibitory effect on *WRN*^–/–^ MSC, as these cells displayed inert AKT signaling. In line with this, mitogenic growth factors such as FGF2, HGF and IGF1 could stimulate AKT phosphorylation in *WRN*^+/+^ but not *WRN*^–/–^ MSCs (Supplementary Fig. 2). We asked whether the AKT pathway could be activated by targeting the upstream SHIP phosphatases. Two potent inhibitors, 3α-aminocholestane (S1) and AS1949490 (S2), have been previously reported to specifically inhibit SHIP1 and SHIP2 respectively (Fig. 3a) [23, 24]. Treatment of WRN-deficient MSC with 1 μM of S1 or 2 μM of S2 could stimulate AKT phosphorylation at both Thr308 and Ser473 residues. Accordingly, the downstream AKT target mTORC1 was activated (p-mTORC1) by S1 or S2 (Fig. 3b). Concordant with the AKT activation, cell proliferation was found to be significantly increased (Fig. 3c, d). While S1 and S2 individually could stimulate AKT pathway and cell proliferation, combined treatment with both inhibitors (S1 + S2) showed an enhanced effect. We further investigated whether SHIP inhibition could promote MSC clonogenicity by colony formation unit-fibroblast (CFU-F) assay. The result showed that S1 and S2 had beneficial effect on promoting CFU number (Fig. 3e, f). Notably, the combined treatment with both inhibitors in WRN-deficient MSCs could stimulate cells to proliferate and form CFU at a level comparable to WRN-WT MSCs.

### Inhibition of SHIP stimulated expressions of pro-angiogenesis factors

**Fig. 4:**
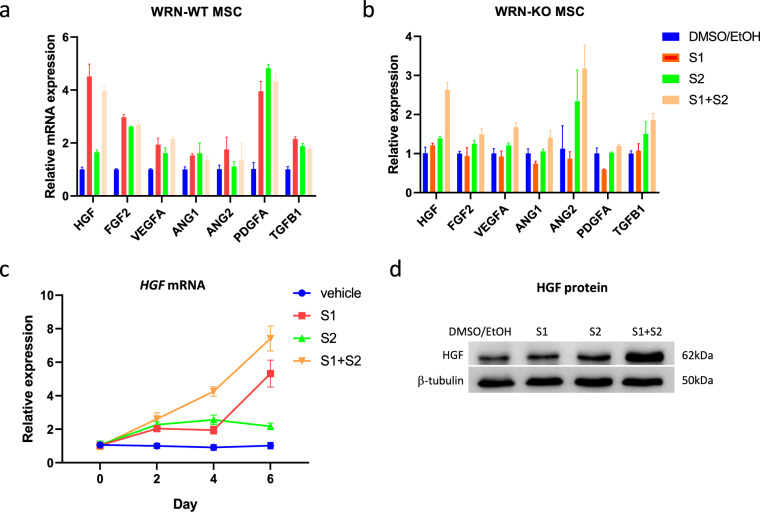
Inhibition of SHIP1 and SHIP2 stimulated expressions of pro-angiogenesis factors. **a** Expression of pro-angiogenesis growth factors in WRN-WT, and **b** WRN-KO MSC treated with S1 and S2. **c** Expression of *HGF* in WRN-KO MSC treated with S1 and S2 for 6 days. **d** Western blot analysis of the intracellular HGF protein level (4 days of treatment)

Next, we examined whether SHIP inhibitors could increase expression of growth factors that promote angiogenesis. In WRN-WT MSC, both S1 and S2 could enhance expression of pro-angiogenic factors including *HGF*, *FGF2*, *VEGFA*, *ANG1*, *ANG2*, *PDGFA*, and *TGFB1* (Fig. 4a). In WRN-KO MSCs, S1 or S2 had minor effect on the expression of these genes. However, combined treatment (S1 + S2) could significantly enhance expressions of *HGF* and *ANG2*, two growth factors that were significantly downregulated in WRN-deficient MSC (Fig. 4b). We analyzed the expression changes of *HGF* in a time course treatment up to 6 days (Fig. 4c). Western blot analysis confirmed the increased level of HGF protein with S1 + S2 treatment (Fig. 4d). These data demonstrated that targeting SHIP could restore some of the paracrine factors for promoting angiogenesis, cell survival and proliferation.

### Inhibition of SHIP ameliorated premature senescence and suppressed IL-6 and IL-8

**Fig. 5:**
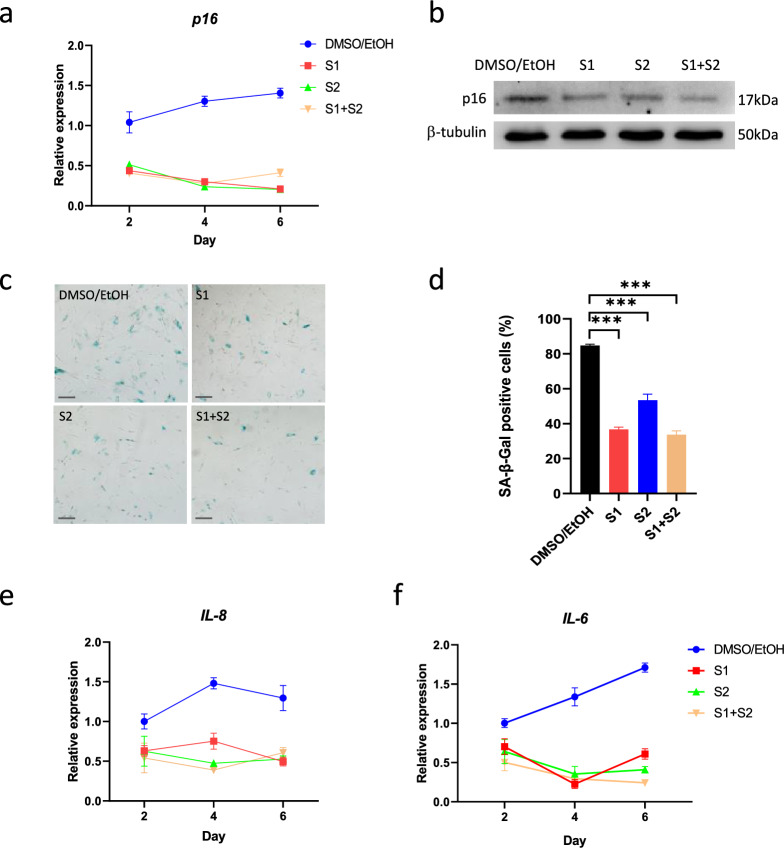
Inhibition of SHIP1 and SHIP2 suppressed IL-6, IL-8 and senescence. **a** Expression of *p16* in WRN-KD MSC treated with S1 and S2 for 6 days. **b** Western blot analysis of p16 protein level (Day 4). **c** SA-β-gal staining for senescent cells in MSC at passage number 9. **d** Quantification of SA-β-gal staining. **e**, **f** Expression of pro-inflammatory cytokines *IL-8* and *IL-6* in WRN-KD MSC treated with S1 and S2. *: *p* < 0.05; **: *p* < 0.01, ***: *p* < 0.001 (by two-sided unpaired Student’s *t*-test). Scale bar: 200 μm

Given that depletion of SHIP1/2 could inhibit senescence, we assessed whether SHIP1 and SHIP2 inhibitors could also lead to similar effects without the need to interfere gene expression. p16 (also known as p16^*INK4A*^) is a cyclin-dependent kinase inhibitor upregulated in aged cells and WS fibroblasts and tissues [25]. We found that the p16 transcripts were significantly decreased when SHIP inhibitors were used to treat WRN-deficient MSCs (Fig. 5a). Western blot analysis showed a significant decrease of p16 protein when both inhibitors were added (Fig. 5b). Moreover, SA-β-gal staining demonstrated a reduction of senescent cells when WRN-deficient MSCs were treated with the SHIP inhibitors (Fig. 5c, d), consistent with the SHIP knockdown result. Encouraged by these results, we further investigated the expression of pro-inflammatory cytokines known to be upregulated in WS serum [26, 27]. We found that both *IL-6* and *IL-8*, two important cytokines that are increased during chronic inflammation, were significantly decreased by SHIP inhibitors (Fig. 5e, f). IL-6 and IL-8 are also associated with SASP evident in senescent cells. These data indicated a beneficial effect of inhibiting SHIP pathway in the amelioration of premature aging in WS stem cells.

### Inhibition of SHIP promoted trilineage differentiation of MSC

**Fig. 6:**
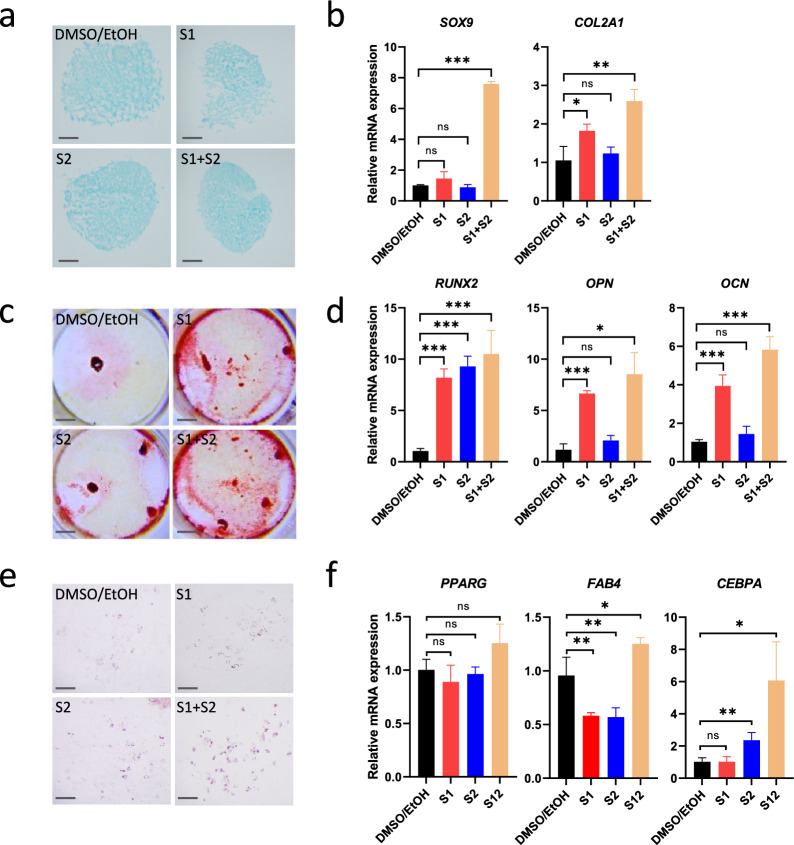
Inhibition of SHIP1 and SHIP2 promoted MSC trilineage differentiation. WRN-KD MSCs (passage number 6–7) were pretreated with S1 and S2 for 4 days before being induced to trilineage differentiations. **a** Alcian Blue staining of the differentiated chondrocytes. The vehicle treated WRN-KD MSC showed loosely aggregated cell mass by the end of the differentiation, whereas S1 + S2 treated cells showed increased coalescence. **b** qRT-PCR analysis of the chondrogenic markers *SOX9* and *COL2A1*. **c** Alizarin Red S staining of the differentiated osteocytes. **d** Expression of osteogenic markers *RUNX2*, *OPN*, and *OCN*. **e** Oil Red O staining of the differentiated adipocytes. **f** Expression of adipogenic markers *PPARG*, *FAB4*, and *CEBPA*. Scale bar: 200 μm for (**a**, **c**) and 3.67 mm for (**b**)

As inhibition of SHIP could activate AKT signaling and prevent WRN-deficient MSC from premature senescence, we next assessed whether treatment with SHIP inhibitors could improve stem cell differentiation. We pretreated WRN-deficient MSCs with SHIP inhibitors for 4 days before the (same number of) cells were replated for differentiations to chondrocytes, oesteocytes and adipocytes. Pretreatment with SHIP inhibitors could enhance the subsequent differentiation to chondrocytes with a better outcome, as indicated by Alcian blue staining of aggrecan deposition in the chondrocyte mass (Fig. 6a). Combined treatment with S1 and S2 significantly increased the expression of *SOX9* and *COL2A1* transcripts, two markers of chondrogenesis (Fig. 6a). Similarly, pretreatment with S1 and S2 enhanced the osteogenesis and adipogenesis and their respective differentiation markers (Fig. 6b, c). Together, these results indicated that inhibition of SHIP promoted differentiation possibly by enhancing stemness of MSC.

### Inhibition of SHIP activated AKT and stimulated growth of WS fibroblasts

**Fig. 7:**
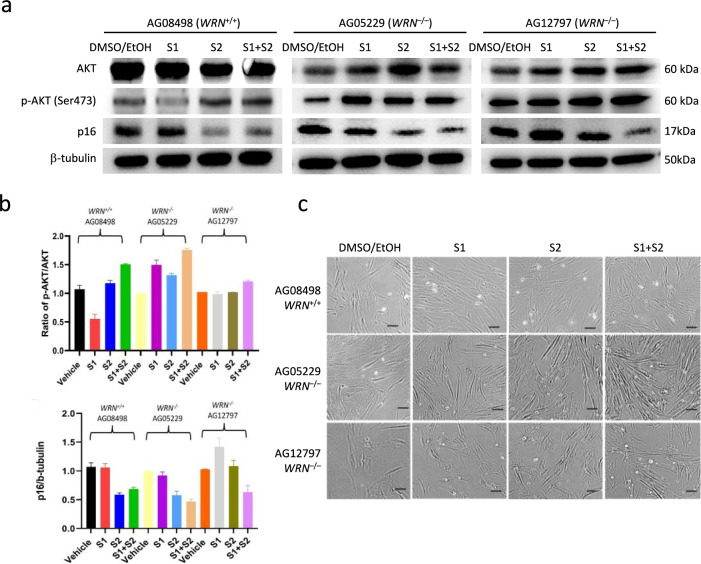
Inhibition of SHIP1 and SHIP2 activated AKT phosphorylation and stimulated growth of WS fibroblasts. **a** Western blot analysis of p-AKT and p16 levels in normal (AG08498) and WS fibroblasts (AG05229, AG12797) treated with S1 and S2. **b** Quantification of p-AKT (normalized by total AKT) and p16 (normalized by β-tubulin) protein levels. **c** Representative bright-field images of the fibroblasts after 4 days of treatment. Scale bar: 200 μm

The above experiments worked on stem cells depleted with *WRN*, the commonly mutated gene in WS. To investigate whether targeting SHIP/AKT signaling pathway is beneficial to primary cells of WS, we obtained WS fibroblasts (AG05229 and AG12797) and normal control fibroblast (AG08498) from cell bank. We treated the cells with SHIP1 and SHIP2 inhibitors for 4 days and analyzed the p-AKT and p16 levels. Consistent with WRN-deficient MSC, WS fibroblasts showed activation of AKT and reduction of p16 after S1/S2 co-treatment (Fig. 7a, b). Treated fibroblasts generally displayed spindle-like morphology typical of active dividing fibroblasts (Fig. 7c). These results suggested that SHIP/AKT signaling pathway also played an important role in the aging of WS fibroblasts.

## Discussion

Aging is regarded as an irreversible biological process, but the process can be accelerated in premature aging diseases such as progeria and progeroid syndromes [28]. Genetic and molecular studies have demonstrated that numerous genes involved in DNA repair, DNA replication, and genome stability maintenance prevent cells from premature aging. WS is a classic adult-onset progeroid syndrome that exhibits multiple aging-associated disorders such as endocrine disorders (diabetes and hypogonadism), vascular diseases, neoplasia, and premature aging phenotypes in connective tissue (skin, bone, and cartilage) [7, 29]. Genetic defects in WS are linked to the RecQ DNA helicase WRN, which unwinds complex DNA structures, such as the G-quadruplex (G4), D-loop, Holliday junction, forked duplex, duplex with 5′-flaps, and triplex [12, 30]. In this study, we revealed that AKT signaling was suppressed in WRN-deficient MSC. We demonstrated that through inhibition of the AKT upstream regulator SHIP, WRN-deficient MSC showed many features of improved stem cell function. These include a higher proliferation rate, fewer senescence cells, higher HGF but less pro-inflammatory cytokine expressions, and better differentiations to chondrocytes, osteocytes, and adipocytes. These results shed light on the potential application of SHIP inhibitors to target premature aging in WS. A preclinical animal model that correctly and precisely mimics WS pathogenesis will be ideal for in vivo tests in the future. In other studies, SHIP inhibition was beneficial in reversing age- and diet-associated obesity and metabolic syndrome in mice [31]. SHIP inhibitors have also demonstrated the ability to stimulate murine hematopoietic and mesenchymal stem cell proliferation and promote microglia to phagocytose dead neurons [23, 32]. However, complete SHIP1 knockout in mice caused myeloproliferative syndrome, whereas SHIP2 deletion conferred resistance to dietary obesity [33, 34]. As the PI3K/AKT signaling pathway is hyperactive in cancers [35], the inhibition of SHIP raises concern about their potential oncogenic effects. Interestingly, however, SHIP could act as an oncogene in some cancers such as hematopoietic cancers. In these cancer cells, the AKT pathway is activated through both PI(3,4,5)P3 and PI(3,4)P2, the latter is the product of SHIP. Both PI(3,4,5)P3 and PI(3,4)P2 are utilized as signaling molecules to maintain a malignant state. Thus, inhibition of SHIP1/2 in blood cell cancers and epithelial cancers was found to compromise cancer cell survival by lowering the PI(3,4)P2 levels and triggering apoptosis [23, 36, 37].

We also observed a downregulation of the TGF-β signaling, as indicated by a consistent decrease in the phosphorylation of signal transduction molecules: ATF2, c-Fos, c-Jun, SMAD1, SMAD2, SMAD4, and SMAD5 (Fig. 1a). TGF-β is a cytokine with diverse functions. It activates the TGF-β pathway, which is known to regulate various biological processes, including embryonic development, wound healing, tissue homeostasis, immune system regulation, chronic diseases (such as fibrosis, inflammation, and cancers), and cellular senescence [38, 39]. The role of TGF-β in MSC aging is somewhat conflicting in the literature. Some reports suggest that TGF-β has a senescence-promoting effect on MSCs [40, 41], while others indicate that TGF-β promotes MSC proliferation without inducing senescence [42–44]. Our findings of downregulated TGF-β signaling in WRN-KD MSCs suggest that TGF-β may act as a senescence inhibitor. The varying effects of TGF-β on MSC aging might be attributed to the different ligands binding to various receptors—there are seven type I receptors and five type II receptors involved in this signal transduction. The non-canonical TGF-β signaling (which is SMAD-independent) activates the PI3K/AKT pathway, leading to the phosphorylation of NF-κB inhibitor kinase (IKK). Activated IKK subsequently promotes the nuclear translocation of NF-κB, which is essential for the activation of genes involved in cell survival, proliferation, metabolism, and immunity. Additionally, TGF-β can activate PI3K either through the kinase activity of TβRI or by recruiting TRAF6. Once activated, PI3K converts PI(4,5)P2 to PI(3,4,5)P3, which triggers the phosphorylation of AKT. Activated AKT (pAKT) has a wide range of cellular effects, including promoting cell survival, growth, proliferation, differentiation, and metabolism [38]. Consequently, the reduced TGF-β signaling in WRN-KD MSCs appears to crosstalk with the diminished AKT signaling, which in turn may impair MSC proliferation, growth, survival, and possibly differentiation.

The question of how the loss of WRN causes increased expression of SHIP protein and downregulated AKT pathway remains elusive. WRN interacts with diverse proteins or protein complexes involved in replication fork stalling, Okazaki fragment processing, proofreading during DNA replication, telomeric DNA replication, and DNA recombination through its exonuclease activity [45]. Moreover, WRN can regulate gene transcription by unwinding G4-rich promoters and thus altering chromatin openness or RNA polymerase II progression [46–48]. We recently determined that WRN unwinds G4 in the *SHOX* promoter and regulates chondrogenesis; a defect in this process contributes to the short stature phenotype in WS [49, 50]. Likewise, WRN may regulate SHIP expression by altering the G4 structures in the promoter or transcribed regions. G4 has been reported to be associated with nucleosome exclusion in open chromatin [51, 52]. The presence of G4 structures in the gene body may allow RNA Pol-II machinery to better transcribe the C-rich antisense strand. A recent G4 profiling (G4access) study revealed numerous G4 structures in WRN-depleted Hela cells [53]. In agreement with this hypothesis, the G4 peaks in WRN-depleted Hela cells were more enriched in introns and 3’-UTR near the transcription termination site at *INPP5D* (which encodes SHIP1) and *INPPL1* (which encodes SHIP2) loci (Supplementary Figs. 3 and 4). A multi-omics approach to survey WRN and G4 occupancy by ChIP-seq and chromatin accessibility by ATAC-seq in MSC may help to answer these questions.

## Conclusions

**Fig. 8:**
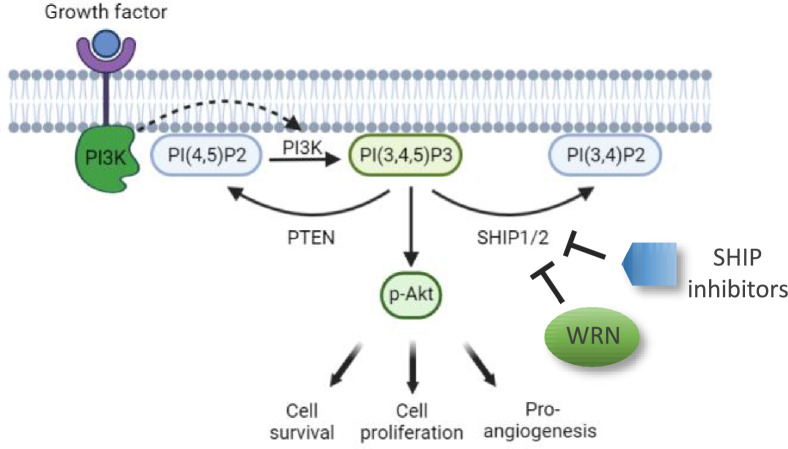
Graphic abstract showing the beneficial use of SHIP inhibitors for the activation of AKT signaling in the absence of WRN helicase. In this model, loss of WRN results in overexpression of SHIP, a phosphatase actively converts PI(3,4,5)P3 to PI(3,4)P2. As a result, AKT phosphorylation is inhibited, and AKT signaling related pathways such as cell survival and proliferation are attenuated

In conclusion, our study suggested that AKT signaling was inhibited in WRN-deficient MSCs. Targeting the SHIP/AKT pathway by SHIP inhibitors is beneficial to WRN-deficient stem cells and fibroblasts, as these cells showed amelioration of premature aging with increased cell survival, proliferation, and enhanced cytokine production and differentiation potential (Fig. 8). Activation of the SHIP/AKT pathway might be applied to improve the senescent phenotype related to WRN mutation in WS patients.

## Materials and methods

### Cell culture

Human umbilical cord-derived mesenchymal stem cells (UC-MSCs) were seeded on culture plate pre-coated with 0.1% gelatin solution at a density of 2 × 10^4^ cells per cm^2^ in MSC culture medium (low glucose DMEM, supplemented with 10% MSC-qualified FBS, 1% GlutaMAX supplement, 1% NEAA solution, 1% Antibiotic–Antimycotic). MSCs were maintained in a 5% CO_2_ incubator at 37 °C with medium change every 2 days and split when reaching 90% confluence. Only cells between passage 4–9 were used for experiments. iMSCs were cultured under the same condition. For human fibroblasts, cells were cultured in MEM medium supplemented with 15% non-heat inactivated FBS, 1% NEAA, 1% GlataMAX and 1% Antibiotic–Antimycotic. Medium was changed every 2–3 days. For 293 T cells, cells were cultured in high glucose DMEM supplemented with 10% FBS, 1% GlutaMax supplement, 1% NEAA solution, 1% sodium pyruvate and 1% Antibiotic–Antimycotic. Cells were split in a ratio of 1:5 to 1:6 when reaching sub-confluence. All medium components were purchased from Gibco.

### Protein array for profiling phosphorylation pathway

Human Phosphorylation Pathway Profiling Array (Raybiotech#AAH-PPP-1–4) was used for screening. First, membranes were blocked in blocking buffer at room temperature for 30 min, then the membranes were incubated with diluted protein samples at 4 °C overnight with gentle orbital shaking. After incubation, the membranes were then rinsed with Wash Buffer I and II for 2 times. Afterwards, detection antibody cocktail solution was applied to the membranes at room temperature for 2 h. Another washing was performed with subsequent horseradish peroxidase (HRP)-labeled anti-rabbit secondary antibody incubation at room temperature for another 2 h. Signals on the membranes were visualized by ChemiDoc (Bio-Rad) using ECL substrate (Bio-Rad).

### Western blot

Cells pellets were rinsed with PBS 2 times were lysed in RIPA lysis buffer (Beyotime) supplemented with protease inhibitor cocktail and phosphatase inhibitors for 20 min on ice and centrifuged at 12,000 rpm for 10 min to collect supernatants. Protein concentration was quantified using Pierce BCA Protein Assay Kit (Thermo Scientific). To denature proteins, 4X Laemmli buffer and β-mercaptoethanol were added to crude protein lysates and heated at 100 °C for 10 min. The mixture was resolved in SDS–Polyacrylamide gel (10–15%) and transferred to PVDF membrane using wet transfer method. Membrane was blocked with 5% non-fat dry milk in PBST for 1 h at room temperature and then incubated with different primary antibodies at 4 °C overnight. Membranes were rinsed with PBST 3 times followed with incubation of secondary antibodies for 1 h at room temperature. Signals were detected by addition of ECL substrate and images were captured by ChemiDoc (Bio-Rad). Antibodies used in this study were listed in Supplementary Table.

### Gene expression analysis by quantitative PCR

Cultured cells were directly lysed in TRIzol reagent (ThermoFisher) and total RNA was extracted. First strand cDNA was transcribed from 500 ng of total RNA using Primescript RT master mix (Takara) by incubating the mixture at 37 °C for 30 min. The resulting cDNA was further diluted to 10 ng/μL. qPCR reaction mix containing 1 μL of cDNA, 0.5 μL of forward and reverse primer mix (10 μM each) and 1X Powertrack SYBR Master Mix (Invitrogen) was prepared and run in ABI QuantStudio 7 Real-time PCR System (Applied Biosystems). Each sample was performed in technical triplicates. Target gene expression was calculated by ΔΔCT method and normalized by internal control gene GAPDH. All qPCR primers sequences were listed in Supplementary Table.

### Construction of plasmids and preparation of lentiviral pseudoparticles

Short-hairpin RNA (shRNA) was cloned in lentivector by ligating annealed oligo duplex to pDECKO_mCherry (Addgene#78534) via a pair of *BsmBI* sites. shRNA sequences used in this study were listed in Supplementary Table 3. The ligated DNA were transformed into the *Stbl3* competent cells. Plasmids were extracted from bacteria and sequenced. To package lentivirus, 293 T was transfected with transgene lentivector and packaging plasmids psPAX2 and pMD.2G at molar ratio 1.5:1:1 using PEI transfection reagent. Culture medium was harvested at 48 h and 72 h post transfection, combined and filtered through a 0.45 $\upmu$m filter. PEG8000 (Santa Cruz) solution was added to form a 5% concentration and incubated at 4 °C overnight. Lentiviral particles were precipitated by centrifuge at 1500 *g* for 30 min, and finally resuspended in cold PBS. The virus stock was stored at − 80 °C for subsequent experiments.

### ${\varvec{\upbeta}}$-Galactosidase assay

Β-galactosidase staining was performed using Senescence β-Galactosidase Staining Kit (Sigma-Aldrich) according to the manufacturer’s protocol. First, cells were rinsed with PBS 2 times and fixed with 1X Fixation Solution in room temperature for 10 min. Then the cells were washed again and incubated with the freshly prepared X-gal substrate solution at 37 °C overnight. Signals were acquired by using Ti2-E Inverted Fluorescence Microscope (Nikon) and analyzed with ImageJ.

### Chondrogenic differentiation and Alcian Blue staining

Chondrogenic differentiation was performed according to published protocol [54]. Before differentiation, MSCs have been treated with SHIP inhibitors or vehicle controls for 4 days. After treatment, MSCs were trypsinized and dispensed at a cell density of 2.5 × 10^5^ cells per well in a polypropylene U-bottom 96-well plate with chondrogenic induction medium (DMEM-HG, 10% ITS + Premix Tissue Culture Supplement, 0.1 µM dexamethasone, 1 µM ascorbate-2-phosphate, 1% sodium pyruvate, and 10 ng/mL TGF-β1). The cells were centrifuged to form pellet. The medium was changed daily and the differentiation lasted for 3 weeks. By the end of the differentiation, total RNA was extracted from the pellets for qPCR analysis. Pellets were also harvested for Alcian Blue staining. After fixation in 4% paraformaldehyde (PFA) solution, pellets were embedded in OCT compound and cryo-sectioned at 10 µm thickness. The sections were stained with Alcian Blue solution in 3% acetic acid for 30 min. Lastly, the sections were dehydrated and mounted on coverslips for imaging.

### Adipogenic differentiation and oil red O staining

Adipogenic differentiation was performed using the StemPro Adiopogenesis Differentiation Kit (Gibco) according to the manufacturer’s instruction. After SHIP inhibitor treatment, MSCs were replated for adipogenic induction. Differentiation medium was changed every 3 days before oil droplets were visualized. Samples were harvested for RNA extraction and Oil Red O staining. To prepare the staining solution, 0.5% Oil Red O solution in isopropanol was mixed with ddH_2_O at a 3:2 ratio and filtered before use. Adipocytes were first fixed in 4% PFA for 30 min and then treated with 60% isopropanol for 5 min. After that, cells were stained with the diluted Oil Red O solution for 15 min. Cells were rinsed with 60% isopropanol to remove residues before being examined under an inverted light microscope.

### Osteogenic differentiation and Alizarin Red S staining

Osteogenic differentiation was performed using the StemPro Osteogenesis Differentiation Kit (Gibco) according to the manufacturer’s instruction. After SHIP inhibitor treatment, MSCs were replated (5 × 10^3^ per cm^2^) for osteogenic induction. Differentiation medium was changed every 3 days for 3 weeks. Samples were harvested for RNA extraction and Alizarin Red S staining. To stain the cells, medium was removed and cells were fixed in 4% PFA for 30 min. After fixation, cells were washed twice by ddH_2_O and stained with 2% Alizarin Red S solution (pH 4.2) for 2–3 min, followed by destaining using ddH_2_O. Cells examined under an inverted light microscope.

### Drug treatment

Normal (*WRN*^WT^) and WRN-deficient (*WRN*^KD^ or *WRN*^KO^) MSCs or dermal fibroblasts were treated with 1–2 μM of SHIP1 inhibitor 3-a-Aminocholestane (S1) or SHIP2 inhibitor AS1938909 (S2) or both for 2, 4 and 6 days. Toxicity test showed that 1 μM of S1 and 2 μM of S2 were the most optimal concentration for 6 days of treatment. DMSO and ethanol used to dissolve S1 and S2 respectively, thus they were used as vehicle control. S1 and S2 were freshly supplemented to the MSC medium at the day of treatment and replenished when medium was changed every other day. Both compounds were purchased from MCE.

### Cell proliferation assay

Proliferation assay was performed using CCK-8 kit (Beyotime) according to the manufacturer’s instruction. MSCs (2 × 10^3^ per well) were plated on gelatin-coated 96-well plate and cultured with or without the SHIP inhibitors added. Cells were cultured and measured in 4 consecutive days. To quantify cell number, 10 μL of CCK-8 solution was added per well and incubated for an hour. Absorbance was measured at 450 nm by SpectraMax i3X plate reader (Molecular Devices) in triplicate.

### Colony formation unit assay

Viable MSCs (0.2 × 10^3^) were seeded on gelatin-coated 35 mm dish. SHIP inhibitors were added to the medium and incubated at 37 °C with 5% CO_2_ for 14 days to form colonies. After colony formation, medium was aspirated and the cells were rinsed with PBS twice. Ice-cold methanol was used to fix the cells for 5 min at room temperature. 2 mL of Wright-Giemsa Stain solution were added to each well and incubated for 10 min, followed by 2 times of PBS washing. The plates were air-dried, and colonies were counted and imaged under a light microscope. Technical triplicate was performed on each sample.

### Statistics

Student's t-test (two-tailed, assuming unequal variance) was performed to compare the difference between two groups. Data were normally presented as the mean value of 3 replicates, and error bars indicate the standard deviations. Statistical significance was determined by the p-value, where “*” denotes p < 0.05, “**” denotes p < 0.01, and “***” denotes p < 0.001.

## Supplementary Information

Supplementary Material 1.

Supplementary Material 2. Increased expression of INPP5Dand INPPL1during replication-induced aging.

Supplementary Material 3. Lack of AKT activation in WRN-deficient MSC.

Supplementary Material 4. G4access profiling in WRN-depleted Hela cells.

Supplementary Material 5. G4access profiling in WRN-depleted Hela cells.

## Data Availability

The datasets during and/or analysed during the current study available from the corresponding author on reasonable request.

## Abbreviations

WS: Werner syndrome
MSC: Mesenchymal stem cell (mesenchymal stromal cell)
SASP: Senescence-associated secretory phenotype
S1: 3α-Aminocholestane (SHIP1 inhibitor)
S2: AS1949490 (SHIP2 inhibitor)
SHIP: SH2-containing inositol 5'-phosphatase
G4: G-quadruplex
