# Supplementary material for: Amelioration of premature aging in Werner syndrome stem cells by targeting SHIP/AKT pathway
Source: Cell Biosci. 2025 Jan 25;15:10. doi: 10.1186/s13578-025-01355-4 (PMC11765919; doi:10.1186/s13578-025-01355-4)
Supplement: Supplementary file 1 — Supplementary Material 1. [file 13578_2025_1355_MOESM1_ESM.docx]

**Supplementary Table 1. Antibodies used in Western Blot**

**Primary antibodies**

| Antibody | Dilution in 5% w/v non-fat dry milk in PBST | Company | Host | Catalog Number |
| --- | --- | --- | --- | --- |
| AKT | 1:1000 | Abclonal | Rabbit | A18120 |
| p-AKT (Ser473) | 1:1000 | Abclonal | Rabbit | AP1208 |
| p-AKT (Thr108) | 1:1000 | Abclonal | Rabbit | AP1259 |
| PTEN | 1:1000 | Cell signaling | Rabbit | 9559 |
| mTOR | 1:1000 | Santa Cruz | Mouse | sc-517464 |
| p-mTOR | 1:1000 | Santa Cruz | Mouse | sc-293133 |
| GSK3β | 1:1000 | Santa Cruz | Mouse | sc-377213 |
| p-GSK3β | 1:1000 | Santa Cruz | Mouse | sc-373800 |
| WRN | 1:1000 | Sigma-Aldrich | Mouse | W0393 |
| SHIP1 | 1:1000 | Abclonal | Rabbit | A0122 |
| SHIP2 | 1:1000 | Abclonal | Rabbit | A10115 |
| p16 | 1:1000 | Abclonal | Rabbit | A0262 |
| HGF | 1:1000 | Abclonal | Rabbit | A1193 |
| GAPDH | 1:1000 | Abclonal | Mouse | AC033 |
| β-tubulin | 1:1000 | Invitrogen | Mouse | 32-2600 |

**Secondary antibodies**

| Antibody | Dilution in 5% w/v non-fat dry milk in PBST | Company | Catalog Number |
| --- | --- | --- | --- |
| Goat Anti-Rabbit IgG (H + L)-HRP Conjugate | 1:5000 | Invitrogen | 1706515 |
| Goat Anti-Mouse IgG (H + L)-HRP Conjugate | 1:5000 | Invitrogen | 1706516 |

**Supplementary Table 2. qPCR primers**

| Targeted gene | Primer Sequences (5’ to 3’) | |
| --- | --- | --- |
| GAPDH | Forward | GTCTCCTCTGACTTCAACAGCG |
|  | Reverse | ACCACCCTGTTGCTGTAGCCAA |
| WRN | Forward | CATTGCTGTGGATGAGGCTCAC |
|  | Reverse | GTAGCAGTAAGTGCAACGATTGG |
| INPP5D | Forward | TGTGACCGAGTCCTCTGGAAGT |
|  | Reverse | GCCTCAAATGTGGCAAAGACAGG |
| INPPL1 | Forward | CTCAAGGAGCTTACGGATCTGG |
|  | Reverse | TGGCTGATACGGTTCTCGTGCT |
| HGF | Forward | GAGAGTTGGGTTCTTACTGCACG |
|  | Reverse | CTCATCTCCTCTTCCGTGGACA |
| FGF2 | Forward | AGCGGCTGTACTGCAAAAACGG |
|  | Reverse | CCTTTGATAGACACAACTCCTCTC |
| VEGFA | Forward | TTGCCTTGCTGCTCTACCTCCA |
|  | Reverse | GATGGCAGTAGCTGCGCTGATA |
| ANG1 | Forward | CAACAGTGTCCTTCAGAAGCAGC |
|  | Reverse | CCAGCTTGATATACATCTGCACAG |
| ANG2 | Forward | ATTCAGCGACGTGAGGATGGCA |
|  | Reverse | GCACATAGCGTTGCTGATTAGTC |
| PDGFA | Forward | CAGCGACTCCTGGAGATAGACT |
|  | Reverse | CGATGCTTCTCTTCCTCCGAATG |
| TGFβ1 | Forward | TACCTGAACCCGTGTTGCTCTC |
|  | Reverse | GTTGCTGAGGTATCGCCAGGAA |
| p16 | Forward | CTCGTGCTGATGCTACTGAGGA |
|  | Reverse | GGTCGGCGCAGTTGGGCTCC |
| IL-6 | Forward | AGACAGCCACTCACCTCTTCAG |
|  | Reverse | TTCTGCCAGTGCCTCTTTGCTG |
| IL-8 | Forward | GAGAGTGATTGAGAGTGGACCAC |
|  | Reverse | CACAACCCTCTGCACCCAGTTT |
| SOX9 | Forward | AGGAAGCTCGCGGACCAGTAC |
|  | Reverse | GGTGGTCCTTCTTGTGCTGCAC |
| COL2A1 | Forward | CCTGGCAAAGATGGTGAGACAG |
|  | Reverse | CCTGGTTTTCCACCTTCACCTG |

**Supplementary Table 3. shRNA sequences used for gene knockdown**

| Targeted gene | Primer Sequences (5’ to 3’) | |
| --- | --- | --- |
| Scramble_shRNA | Forward | GCCTAAGGTTAAGTCGCCCTCG |
|  | Reverse | CGAGGGCGACTTAACCTTAGGC |
| WRN-shRNA | Forward | GAGGGTTTCTATCTTACTAAA |
|  | Reverse | TTTAGTAAGATAGAAACCCTC |
| SHIP1-shRNA1 | Forward | GATTTGAGGGTGGAGATATAG |
|  | Reverse | CTATATCTCCACCCTCAAATC |
| SHIP1-shRNA2 | Forward | GCTCATTAAGTCACAGAAATT |
|  | Reverse | AATTTCTGTGACTTAATGAGC |
| SHIP2-shRNA1 | Forward | CCACCCAAGAACAGCTTCAAT |
|  | Reverse | ATTGAAGCTGTTCTTGGGTGG |
| SHIP2-shRNA2 | Forward | GACTACCTGAAAGGCAGCTAT |
|  | Reverse | ATAGCTGCCTTTCAGGTAGTC |
