# Supplementary material for: Amelioration of premature aging in Werner syndrome stem cells by targeting SHIP/AKT pathway
Source: Cell Biosci. 2025 Jan 25;15:10. doi: 10.1186/s13578-025-01355-4 (PMC11765919; doi:10.1186/s13578-025-01355-4)
Supplement: Supplementary file 2 — Supplementary Material 2. Increased expression of INPP5Dand INPPL1during replication-induced aging. [file 13578_2025_1355_MOESM2_ESM.pdf]

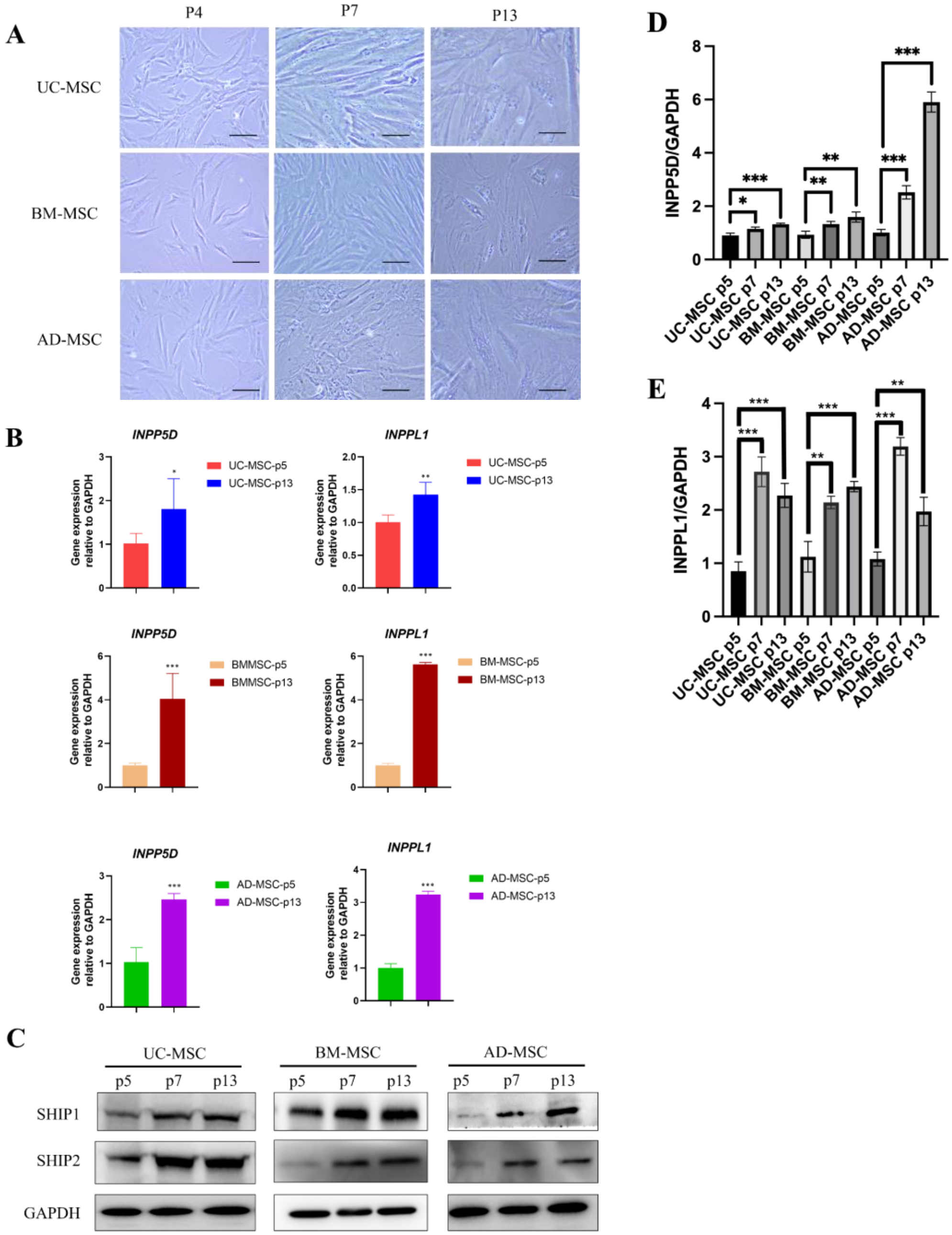

Supplementary Figure 1. Increased expression of INPP5D (SHIP1) and INPPL1 (SHIP2) during replication-induced aging. (A) Cell morphology of MSCs at different passage numbers. UC-MSC: umbilical cord derived MSC; BM-MSC: bone marrow derived MSC; AD-MSC: adipose tissue derived MSC. (B) Expression of INPP5D and INPPL1 in p5 and p13 MSCs. (C) Western blot analysis of SHIP1 and SHIP2 proteins at different passage numbers of MSCs. (D, E) Quantification of the results in C. *: $p < 0.05$; **: $p < 0.01$, ***: $p < 0.001$ (by two-sided unpaired Student's *t*-test).
