# Supplementary material for: Amelioration of premature aging in Werner syndrome stem cells by targeting SHIP/AKT pathway
Source: Cell Biosci. 2025 Jan 25;15:10. doi: 10.1186/s13578-025-01355-4 (PMC11765919; doi:10.1186/s13578-025-01355-4)
Supplement: Supplementary file 3 — Supplementary Material 3. Lack of AKT activation in WRN-deficient MSC. [file 13578_2025_1355_MOESM3_ESM.pdf]

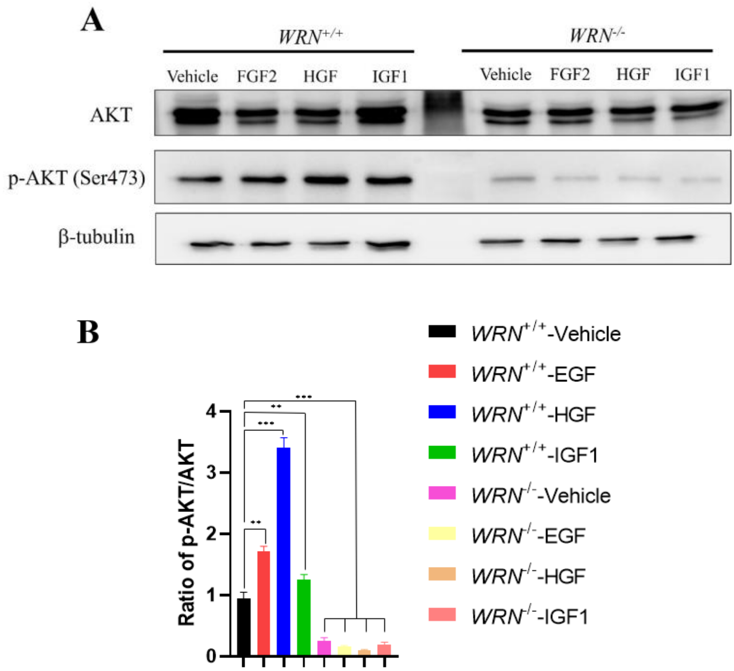

Supplementary Figure 2. Lack of AKT activation in WRN-deficient MSC. (A) *WRN*$^{+/+}$ and *WRN*$^{–/–}$ MSCs were treated with different growth factors for an hour to activate AKT pathway. Western blot analysis showing the levels of p-AKT (Ser473) and total AKT. (B) Quantification of A. *: $p < 0.05$; **: $p < 0.01$, ***: $p < 0.001$ (by two-sided unpaired Student's *t*-test).
