# Supplementary material for: Amelioration of premature aging in Werner syndrome stem cells by targeting SHIP/AKT pathway
Source: Cell Biosci. 2025 Jan 25;15:10. doi: 10.1186/s13578-025-01355-4 (PMC11765919; doi:10.1186/s13578-025-01355-4)
Supplement: Supplementary file 5 — Supplementary Material 5. G4access profiling in WRN-depleted Hela cells. [file 13578_2025_1355_MOESM5_ESM.pdf]

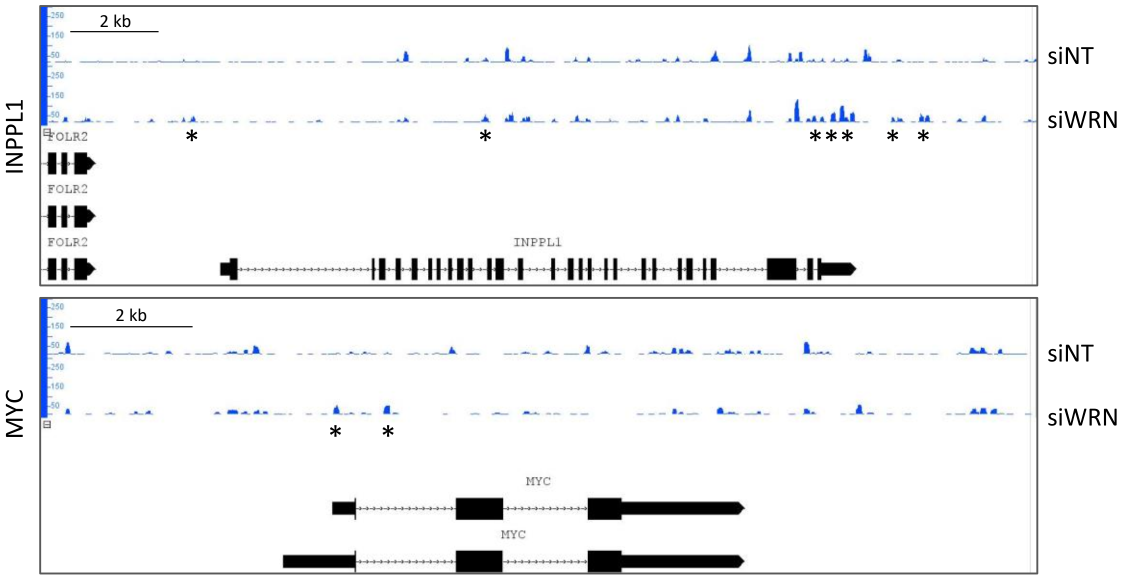

Supplementary Figure 4. G4access profiling in WRN-depleted Hela cells (siWRN). Unprocessed data were retrieved from GEO (GSE187007) and displayed in IGB. Genomic regions at *INPPL1* (SHIP2) and *MYC* were shown. The *MYC* promoter contains a G4 sequence that is known target of WRN. Genomic regions with increased G4 peaks were highlighted with asterisks.
